# Exploring the Factors That Impact Recruitment and Retention of Pediatricians in Irish Community Hospitals Through the Attitudes of Trainees and Physicians-in-Practice

**DOI:** 10.1177/23821205241285599

**Published:** 2024-10-08

**Authors:** Lydia Healy, Michael J O'Grady, Nigel Fancourt, Briseida Mema

**Affiliations:** 1 Department of Critical Care Medicine, Hospital for Sick Children, Toronto, Ontario, Canada; 2 150588 Department of Education, University of Oxford, Oxford, UK; 3 Department of Paediatrics, 57998 Regional Hospital Mullingar, Co. Westmeath, Ireland; 4 Women's and Children's Health, 37438 School of Medicine, University College Dublin, Dublin, Ireland; 5 Department of Pediatrics, 12366 Temerty Faculty of Medicine, University of Toronto, Toronto, Ontario, Canada

**Keywords:** pediatrics, postgraduate education and training, sequential analysis, survey, recruitment, generalist, resuscitation, neonatology

## Abstract

**BACKGROUND:**

Difficulty attracting physicians to work in rural and remote areas is a worldwide problem. Specific to pediatrics, barriers to recruitment include burdensome on-call rosters, a lack of career opportunities for partners and professional isolation.

**METHODS:**

To examine attitudes to working in a community hospital in Ireland, a mixed-methods sequential analysis approach was undertaken. Pediatricians-in training (70) and attending community pediatricians (25) completed surveys. Six semistructured interviews were used to triangulate survey results.

**RESULTS:**

Most trainees planned to stay in Ireland (66/70), with five (eight%) stating that a career in a community hospital was their first preference. Personal factors such as a partner's career prospects and closeness to family and friends were the most important deterrents to working in a community hospital for trainees. Both trainees and attendings were concerned regarding professional isolation. Trainees were concerned about the poor reputation of community units. This converged with attendings feeling their role was not adequately respected, even though their job had more variability and exposure to emergencies, with less support, than working in a large center. Both groups agreed that targeted postgraduate training pathways and better training opportunities within Ireland were the best way to improve recruitment. Financial bonuses were not highly ranked as potential incentives. Motivators for considering a career in community units included the desire to make an impact and to build something new. Concerns about job satisfaction, professional recognition, and limited support for service development were prevalent.

**CONCLUSION:**

This study reveals critical challenges and motivators influencing the shortage of pediatricians in Irish community units. Addressing these issues requires a multifaceted approach, incorporating targeted training, support structures, and recognition to enhance recruitment and retention in these underserved areas. Insights from the Irish context could be applied to improve recruitment and retention of pediatricians in regions with similar contexts.

## Introduction

Many countries report difficulty recruiting physicians to rural and remote areas.^1–5^ This study focuses on the situation in small, community pediatric units in Ireland. Available data demonstrate few pediatric trainees are interested in a career in such units,^6^ but the reasons underpinning this are poorly appreciated. These reasons are explored in this study.

Pediatric care in Ireland is organized in three main levels; tertiary pediatric hospitals with intensive care are in Dublin on the east coast, with regional referral pediatric units within three large teaching hospitals in the major cities. The remainder of pediatric care is provided in thirteen “local” (hereto called “community”) units in smaller towns.^7^ The Republic of Ireland has a medium population density of 73 people per km^2^. Thirty-three percent of the population live in the catchment area of community units,^8^ where pediatricians provide coverage for inpatients, emergency presentations and “Level One” neonatal units (>32 weeks and >1500 g) with suboptimal access to tertiary pediatric care.

Available reports show 45 (17%) of 262 Irish attending posts in pediatrics are unfilled or filled temporarily, mostly in community units in more rural settings.^9^ In 2016, only two of 92 senior Irish pediatric trainees wanted to pursue a career in these units.^6^ Elsewhere, where remoteness and rurality are exacerbated by large landmasses, research has found that burdensome on-call commitments, lack of career opportunities for partners, and professional isolation are barriers to recruitment in remote pediatric units.^10,11^ Professional isolation included lack of interprofessional support for clinical care and lack of vacation coverage. Examining the Irish community context, achieving and maintaining competency in resuscitation, particularly neonatal procedures, is a specific training concern. This aligns with current evidence that proficiency in intubation, a key element of neonatal resuscitation, is increasingly not acquired during pediatric training programs and is difficult to maintain in practice.^12–15^

A collaboration of experts from countries with low population density created a framework to improve physician recruitment to remote areas.^16^ Suggested educational interventions involved promoting positive rural training experiences, targeted postgraduate training, and continuous professional development. Other key principles were recruiting physicians with “generalist” skills rather than “specialists filling gaps”, and integration of physicians within communities.

Building on the existing evidence on physician recruitment in remote areas, we wanted to understand further the contextual factors that impact the shortage of pediatricians in Ireland's community units specifically.^1–6,10,11,16^ The aims of the study were to explore the attitudes of the future Irish pediatric workforce (trainees) and the current workforce (attendings) toward working in community units. These perspectives are then used to the understand recruitment and retention issues affecting the units.

## Methods

We employed a mixed-methods sequential exploratory approach, combining quantitative data from cross-sectional surveys with qualitative data from comments and interviews. The Royal College of Physicians of Ireland (RCPI) and the University of Oxford provided ethical approval. Written consent was obtained from participants. The reporting of this study conforms to CROSS and SRQR criteria (Supplemental Digital Content 1 and 2).^17,18^

### Participants and recruitment

Survey participants were recruited purposefully through RCPI email lists and professional social media groups forwarding a standardized invitation letter. The inclusion criteria were (1) senior pediatric trainees enrolled on the RCPI Higher Specialist Training Pathway^19^ and (2) attending pediatricians for whom most of their current practice was in a community unit in Ireland. Regarding interviews, all interested participants who satisfied the inclusion criteria and consented after receiving information about the study's background were included.

### Survey design

The survey questions were created by LH and MOG. LH was a pediatric trainee in Ireland. MOG is a pediatrician working in a community unit, involved in leadership and teaching in postgraduate pediatric training in Ireland, and experienced in survey methodology. The two groups—trainees and attending pediatricians—completed different surveys. The trainee survey explored decision-making for future careers and potential motivators for a job in the community units. The attending survey examined factors that impacted their decision to pursue a career in a community unit, factors that may improve or hinder recruitment and retention, and their perceptions of their practice context*.* The surveys were created using the principles of survey methodology and informed by the existing literature.^10,11,16,20^

Surveys were piloted with 2 participants (a trainee and an attending) to ensure clarity of the instructions and functionality of the website (https://www.onlinesurveys.ac.uk). Feedback resulted in minor adjustments to instructions*.* Both surveys are included in supplemental digital content (SDC3 and 4). The surveys were conducted during the period May to August 2023. The authors aimed for a response rate of 65% for trainees based on reported standards in medical education literature.^21,22^ Calculating the response rate for attending pediatricians was not possible, as accurate data on the total numbers are not available in published workforce reports.^7,9,23^ While the trainee survey included specific questions on both “push” and “pull” factors for working in a community unit, the presentation of results focuses on the former as few trainees planned to pursue a career in a community unit.

### Interviews

LH completed the interviews in August 2023 after the survey analysis. The purpose of the interviews was to explore and further explain the survey data and triangulate the findings. Interviews followed a semistructured format, with two separate guides for trainees and attendings. The trainees’ interviews addressed decisions influencing career plans, attitudes to working in a community unit and how prepared they felt for community work after completing training. Attending pediatricians’ interviews focused on perceptions regarding the differences between practicing in an academic center versus a community unit and the preparation of trainees for this role. Additionally, attendings were asked about potential suggestions to improve recruitment and retention.

### Statistics and data analysis

Quantitative survey data were analyzed using descriptive statistics in Microsoft Excel. Interviews were audio-recorded, transcribed verbatim, and de-identified. The transcripts were analyzed and coded inductively by LH and BM. BM is an academic critical care physician and education researcher with experience in qualitative methodology. A thematic analysis was conducted with open coding, iterative analysis, and constant comparison with coding, searching, reviewing, defining, and naming themes.^24,25^ Free-text data from both groups of surveys were included in the thematic analysis. To remain open to new interpretations as the data were iteratively analyzed, LH and BM individually coded data before engaging in dialogue and re-examining the transcripts to resolve differences in interpretation. In line with a sequential explanatory design, the qualitative results were used to inform and enrich interpretations of the quantitative data.^26^ Considering that (1) our goal was to describe the factors that impacted recruitment and retention rather than theory building, (2) survey comments were combined with interview data, and (3) in-depth reflections through interviews generated rich data, we reached thematic sufficiency after six interviews.

## Results

There was a 47% response rate, with 70 out of 147 eligible trainees completing the survey. Twenty-five attending pediatricians responded to the survey. Three attending pediatricians and three trainees were interviewed. All trainees had completed at least five years of pediatric training. The attendings interviewed had been in their role for between one and 15 years. The interviews lasted 30 to 90 min. Interviewees are identified as T1-3 (trainees) and A1-3 (attendings). First, we present the survey data regarding trainee career preferences. An integrated analysis of survey and qualitative data (free-text comments and interviews) follows this.

### Trainees career preferences

None of the trainees wanted to pursue a career as a general pediatrician without subspecialization, and 27 (39%) of 70 were interested in general pediatrics with an additional special interest. Thirty-five (50%) intended to become subspecialists, with neonatology being the most popular choice (13/35; 37%). Eight trainees (11%) remained undecided.

Most trainees (66/70; 94%) planned to work in the Republic of Ireland after training. Among those, the majority (35/66; 53%) preferred to work in a tertiary unit in Dublin, 13 (20%) preferred a maternity unit, another 13 (20%) preferred a regional unit, and only five (8%) chose to work in a community unit as their first preference. Twelve of 66 trainees (18%) stated a career in a community unit was their second preference, while it was third preference for 31 (47%) of 66. Eighteen trainees (27%) considered a local unit as their very last option. Half of the trainees reported they would consider working in a community unit, regardless of initial preference.

### Factors that deter trainees’ from working in a community unit

**Figure 1.**
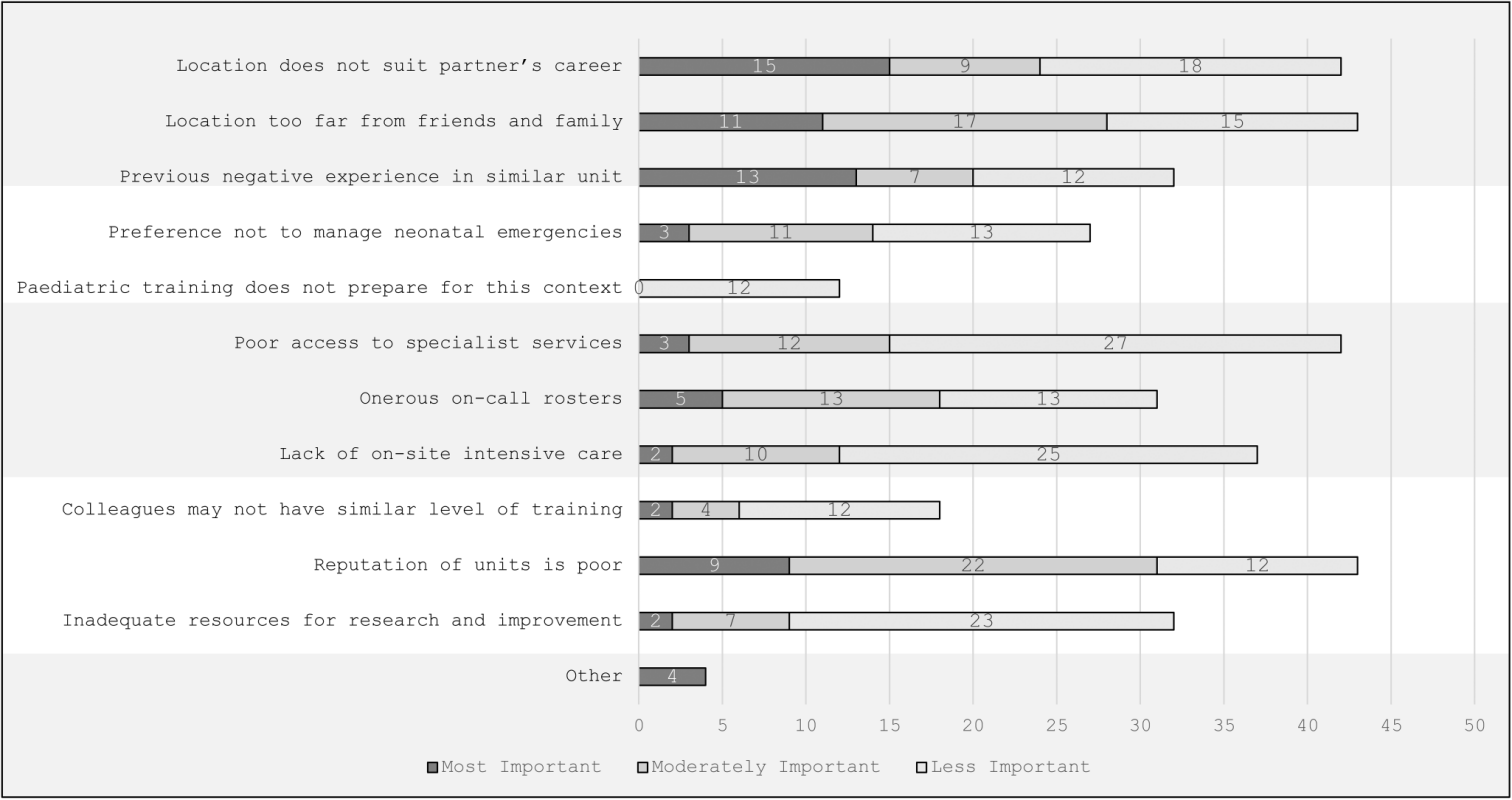
Factors that discourage pediatric trainees from pursuing a career in a community hospital.

Trainees were asked to rank as many items as desired from a list of potential deterrents to working in a community pediatric unit. The list included personal, educational, environmental, and community factors (Figure 1).

Trainees ranked personal factors as the most significant deterrents to working in a community unit; these were a partner's career prospects (15/70, 21%) and closeness to family and friends (11/70; 16%). The qualitative data shed more light on this; T1 reflected on how centralized training leads to trainees establishing roots in cities; “*predominantly our three final years of training, if we do it in Ireland, are in Dublin….And they’re the years when people often start having families*.” T2 elaborated “*I think the days of a man who has a family who can move around with him kind of freely…. that doesn't really exist to the same degree anymore. So, you know, people do have lives…. they have families that have settled*,” indicating how contemporary families with both partners in employment are more impacted by these factors.

Regarding educational factors, 13 (19%) of 70 of trainees said a previous negative experience during their rotation in a community unit was the most important reason not to consider that career. Qualitative data indicated trainees were influenced by aspects of the environment and professional community of the units during their training rotations (see below), rather than purely educational factors. Investigating preparedness for practice at the end of training, we found the following: not wanting to manage neonatal emergencies was ranked as the most important deterrent by three (4%) of 70, moderately important by 11 (16%) of 70 and less important by 13 (19%) of 70 of trainees. The option “training had not prepared me for the role of pediatrician in a community unit” was not selected as most important by any trainee. Although 43 (61%) trainees said they would be comfortable to lead neonatal resuscitations, the sequential qualitative analysis confirmed trainees felt their skills were inadequate. T2 said that following their neonatal rotation: “*I felt I had learnt some skills, but had I learned them to the extent to be a competent consultant* [attending]?” This influenced them to prefer a career in a unit without neonatal responsibilities. Survey comments and interview data converged with linking additional neonatal exposure during training to an increased willingness to work in a community unit.

Environmental factors were frequently selected as disincentives to working in a community unit. Trainees ranked these options as moderately or less important. These included poor access to specialist services (selected by 42/70, 60% overall), lack of on-site intensive care (37/70, 53% overall), and burdensome on-call rosters (31/70, 41% overall). Data from interviews and comments confirmed that call rosters and access to specialist and intensive care services were secondary deterrents to trainees considering this career path.

Regarding factors relating to the professional community, trainees indicated low concern about potential colleagues’ level of training (second least selected factor overall; 18/70; 26%). Instead, qualitative data emphasized the overarching importance of community and culture in a unit for trainees considering this career path. T3 said “*You might be dealing with a lot of locum staff who aren't familiar, and I would imagine that gets a little bit tiring and puts a bit more pressure on the permanent consultants* [attendings]*, so it could be isolating*,” showing concern that recruitment issues can lead to professional isolation when full-time staff in a community unit become overburdened with administrative and leadership duties. Both sources of qualitative data showed trainees felt joining a unit where colleagues’ culture was committed to improvement was a motivator, while some expressed concern regarding recruiting well-trained junior staff. Poor unit reputation was ranked most important by nine (13%) of 70 and selected by 43 (61%) of 70 trainees overall. Qualitative data on this topic elaborated on difficulty recruiting permanent staff, perceived burnout among attendings in community units, and concern about certain units not following guidelines. Thirty-two (46%) of 70 respondents selected lack of resources for service improvement as a deterrent, mostly in the less important category. Qualitative data revealed two sides to this factor. T1 said “*The ability to improve the services we provide to sick kids would be a big motivation…. in comparison with…. *[working in a department in tertiary center] … *you have much bigger scope to improve things or to have an impact and make changes for the better in a unit*,” seeing the opportunity to build and create as a motivation to work in a smaller unit. Meanwhile, other trainees referenced their experiences working in under-resourced clinics as a push factor.

### Trainees assess potential interventions to improve recruitment to community units

**Figure 2.**
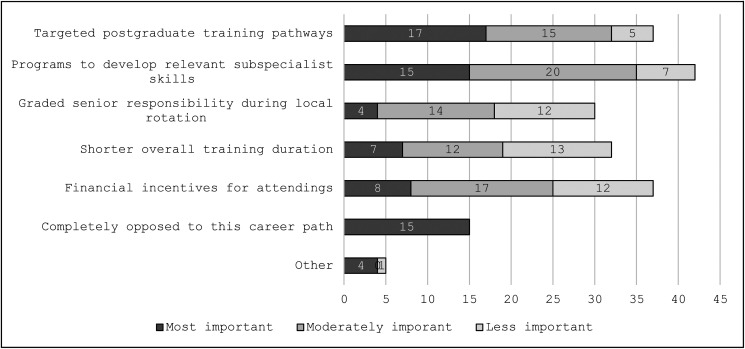
Trainees’ perspective on potential motivators to work in a community unit.

Trainees were invited to rank as many factors as possible that would motivate working in a community unit (Figure 2). The highest selected solution was targeted postgraduate training pathways followed by improving training opportunities in Ireland for skills that would be relevant in the community setting. Currently, the RCPI offers formal Pediatric subspecialty training in neonatology and cardiology only. Outside these subspeciality pathways, the system similarly lacks other opportunities to prepare pediatricians to work in community units. Potential financial incentives were considered most important by only eight (11.4%) out of 70 trainees. Instead, qualitative data showed they perceived a lower cost of living outside major urban areas as a potential incentive. Fifteen (21%) trainees reported that nothing would attract them to work in a community unit.

### Attending pediatricians’ reflection on the nature of work in community units

**Figure 3.**
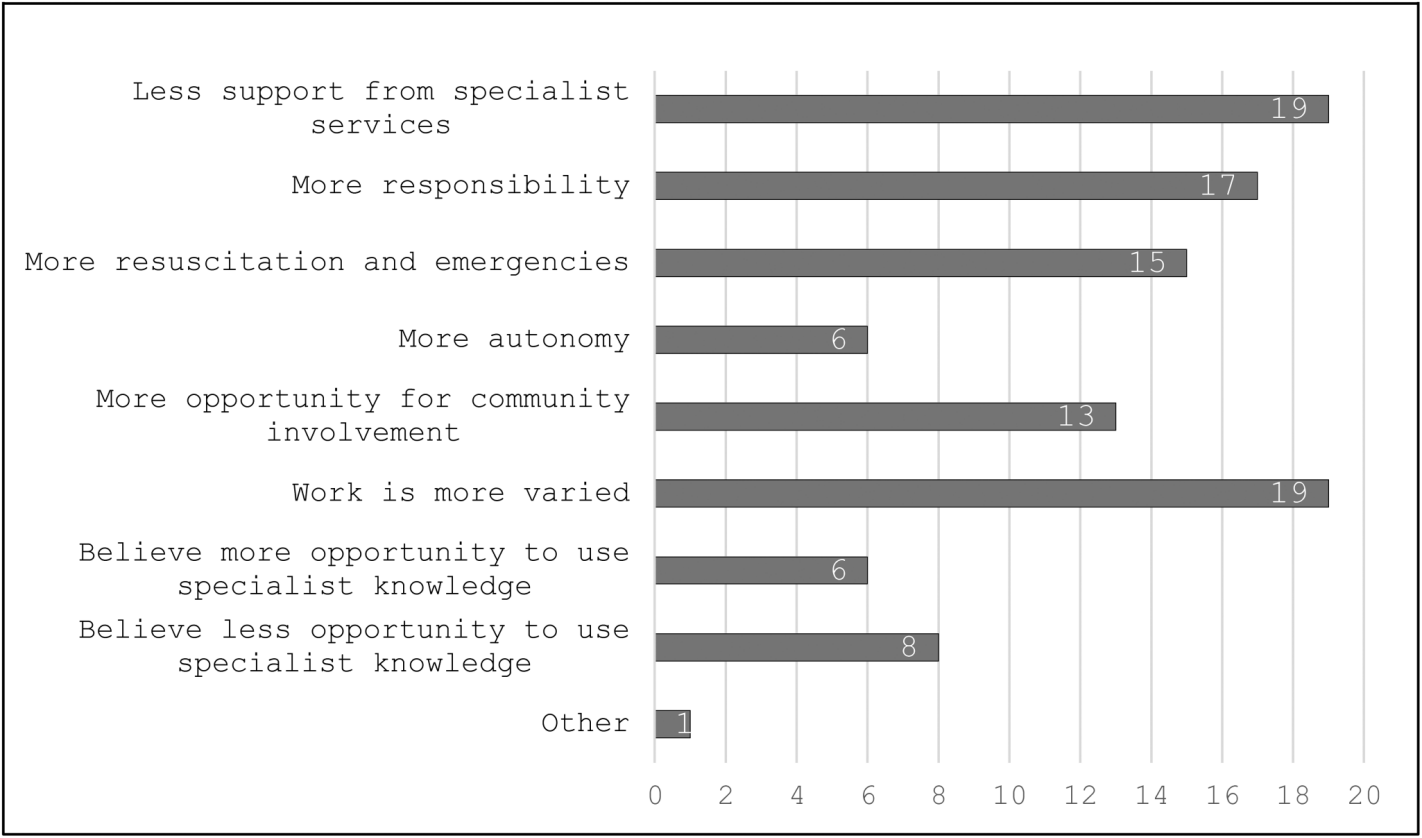
Attending pediatricians’ reflections on differences between community units and academic centers.

Attending pediatricians were asked to reflect on contextual differences between academic and community units (Figure 3). Approximately 75% reported that their work had more variability, they had less support from specialist services and had more responsibility. Fifteen (60%) agreed that there was more exposure to resuscitation and emergencies. Elaborating on this topic, A3 said “*If a sick child comes in, I don’t just have the luxury of calling PICU* [pediatric intensive care]* …. I am PICU until the baby is transferred. I think that your role as pediatrician outside Dublin has a lot more stabilizing of very unwell children*.” A2 thought specific training was needed to deal with the breadth scope in a community unit:

“I have seen that many [attending colleagues] when they got a post in a small hospital equivalent to this one, many of them quit because they were not able to cope with the stress of being exposed to so much….Or you are trained to cope with that, and you gain confidence because you have the proper skill set that you need for that place… Or it's going to be really difficult, and people will be looking all the time to move to another place.”

### Attending pediatricians assess potential interventions to improve recruitment to community units

**Figure 4.**
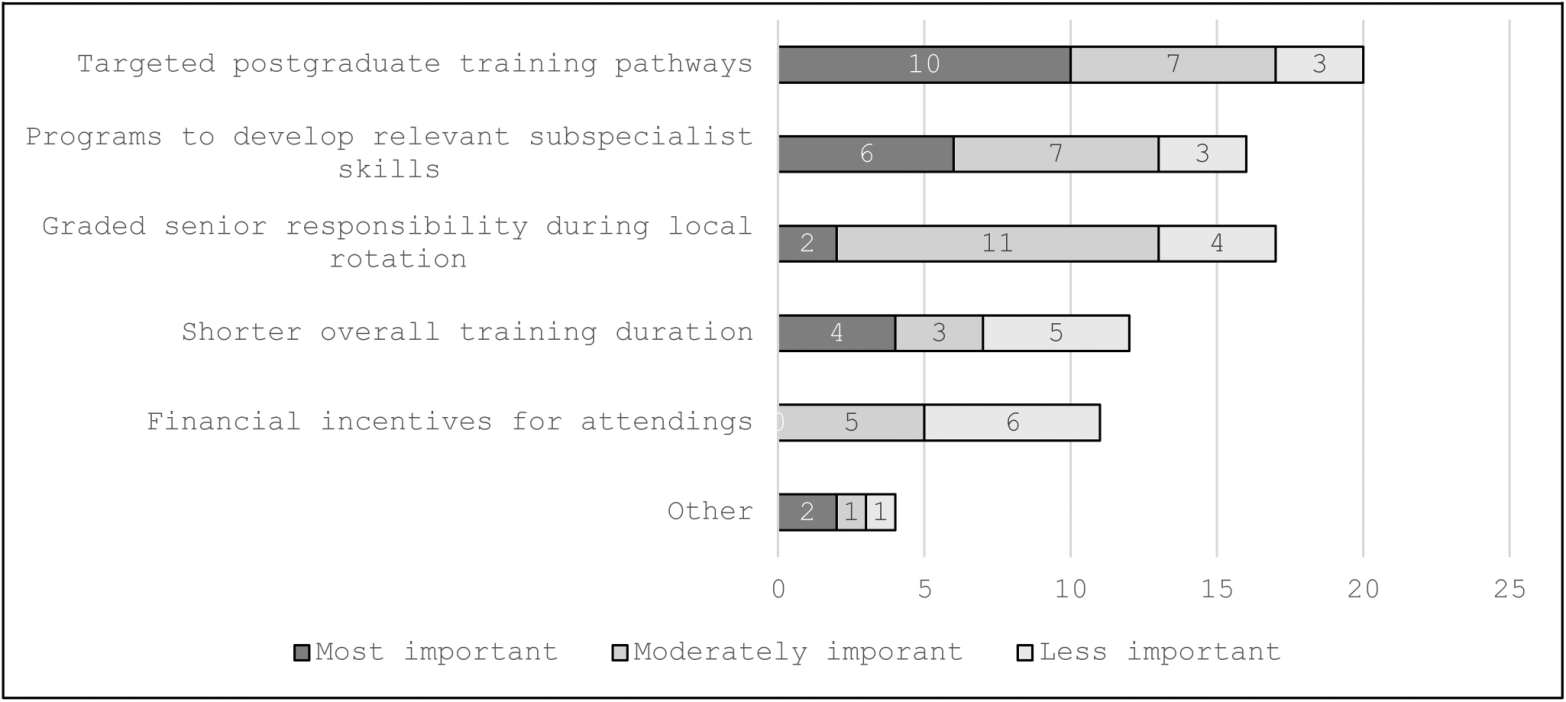
Attendings’ perspective on potential motivators to work in a community unit.

Figure 4 shows how attendings ranked potential suggestions to attract and motivate more trainees to pursue a career in a community unit. While the survey questions focused on interventions aimed at recruiting newly certified attendings, the interviews and free-text comments generated more reflections relating to retention. Converging with trainee responses, practicing pediatricians advocated for training pathways and opportunities that prepare someone to function well in a community setting.

When reflecting on training interventions, attendings cited the difficulties experienced by current trainees in attaining neonatal skills. A2 added “*We cannot fix the problem of lack of skills in intubation. Let's change the rules. Go for an LMA* [laryngeal mask airway],” advocating to replace skills that are difficult-to-acquire and maintain, such as intubation, with more achievable alternatives. An additional educational theme emerged in qualitative data: that attendings perceived how difficult it was in the current system to maintain competence in key skills. A1 said “*It feels like if you could design a system where you’re going to make it really difficult for people to actually do the best job in the world and not support them in any way to maintain their skills or plan for that, you design the system that we currently have in small hospitals*.” They invoked how infrequent exposure to critical scenarios creates a barrier to maintaining acute skills. Attending pediatricians highly desired continuing education provided by experts for infrequently encountered clinical scenarios.

Another theme that emerged was the need for improved recognition of the role fulfilled by these attending pediatricians. A3 suggested “*snobbery*” prevented trainees from pursuing a role in community units, while another attending said a “*superior attitude* [toward community units] *is fostered in the bigger hospitals*.” A2 expressed “*… if one were to erase* [the community units] *from the system and you get the people in the big hospital working without the help or the triage of the others, the system will collapse completely*,” revealing the paradox between the crucial role that these clinicians played and the value that the system placed on them.

Many suggested that appropriate clinical support could promote recruitment and retention. One said “*24-hour retrieval is essential, but progress is very slow with this*,” while others suggested that improving collaboration between pediatricians, surgeons, anesthesiologists, and emergency physicians within community units would optimize care for complex cases. Currently neonatal transport is available “24/7”, while pediatric retrieval is daytime only. Another recurring theme in the data was the difficulty in recruiting trainees with an adequate skill set. Currently in Ireland, a significant proportion of trainees supporting attendings in community units are not part of a recognized postgraduate training program.^9^

Furthermore, attending pediatricians regretted the lack of research and quality improvement support. A2 expressed, “*Here, if people want to pursue research activity there are a lot of barriers. We are trying to implement a project, but it is so difficult. There's no culture. And research is a patient right.*” Other comments highlighted similar difficulties with clinical and academic growth.

No attending selected financial incentives as most important. The comments and interviews focused on personal motivators and quality of life in a rural setting. Repeatedly, the frequency of call commitments was referenced as a barrier to recruitment and retention.

**Figure 5.**
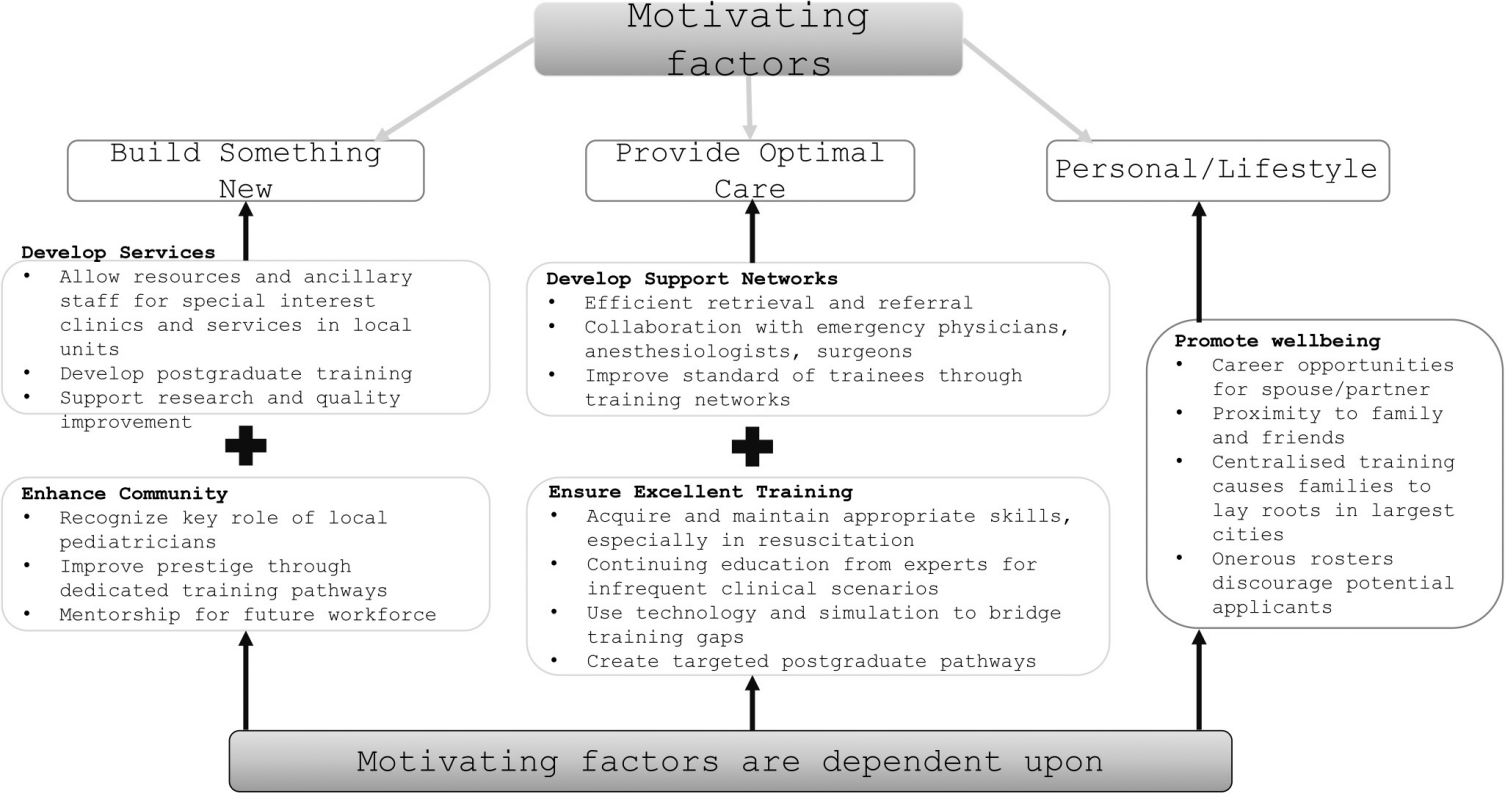
How to attract and support pediatricians to work in a community unit.

Figure 5 summarizes the themes that emerged from survey comments and interviews with graduating trainees and attending pediatricians in these community units.

## Discussion

Our study confirms that a minority of Irish pediatric trainees intend to pursue careers in community units. We shed light on the difficulties experienced by attending pediatricians in these units in Ireland and how they impact trainees’ career choices. We suggest strategies to improve recruitment and retention aligned with the literature and framework by Abelsen et al.^1–3,5,10,11,16^

This study's findings converge with international literature when exploring recruitment issues. Our participants cited personal factors as most important when considering work in community units and they highlighted their partner's career opportunities as a deciding factor. This could be addressed through decentralization of training, allowing families to build lives outside major cities earlier in their careers.

Further exploration of recruitment issues highlighted that smaller units with less on-site support require “extended generalists” and suggested targeted postgraduate training for achievement of competence.^16^ Correspondingly, Irish trainees and pediatric attendings thought targeted training would improve recruitment to community units. As stated above, Ireland has no targeted pathway to train future pediatricians to work in community units. Ireland's national vision for pediatric care states that pediatricians working in community units should cultivate subspecialist skills in high-utility areas such as cardiology, respirology, or endocrinology.^7^ Traditionally, Irish doctors have gone abroad for such subspecialist training.^27^ Both trainees and attendings thought more local training opportunities in these areas would improve recruitment to community units. While some progress has been made such as a formal opportunity in cardiology,^28^ additional options as suggested by the vision for pediatric care are lacking.^7^ Similarly, trainees and attendings agreed that more exposure to neonatology would benefit those working in a community unit but no formal opportunities exist for this.

Retention and recruitment are often intertwined, and we examined working conditions in community units and their impact on both. Many trainees stated that a negative experience during their community rotation discouraged them from pursuing a community career path. Professional isolation is described as a deterrent to recruitment in the literature.^10,11^ This was appreciated broadly in the Irish context. Trainees were worried about overreliance on locums with increased pressure on permanent staff. Attending pediatricians were overwhelmed by call burden, neonatal resuscitation responsibilities, and limited access to intensive care and retrieval services. Therefore, implementing a “24/7” pediatric retrieval should therefore be a pivotal goal to supporting the workforce in these units.

Both groups desired support for research and quality improvement. Trainees saw the opportunity to improve services was a potential pull factor to work in community units. Provision of dedicated funds for research, quality improvement, and continuous medical education initiatives should improve recruitment and retention. A specific finding from our study relating to pediatrics is that attendings in community units wanted ongoing education from experts in resuscitation. The broader literature increasingly recognizes the importance of maintaining such competence at the attending level and there are successful blueprints for implementing such competency training.^29^ Lastly, our study highlights a new finding not previously reported in the literature: attending pediatricians in these units feel undervalued for their work. Attendings struggled with the paradox of what they were asked to manage—more challenging cases with less support and resources—while holding a less respected role within the wider pediatric community. Programs that target appreciation and recognition of the pediatrician in community units could support retention and recruitment.

This study sheds light on the differences between generations of pediatricians. Trainees were most concerned about personal factors while attendings focused more on working conditions and supports. Literature in Ireland and elsewhere has shown that younger doctors are more concerned about work–life balance.^30,31^ Interestingly, while trainees said they were deterred from working in units with a poor reputation, the attending pediatricians working in these units felt inadequately respected by trainees and pediatricians in larger centers.

This study has certain limitations, including the response rate that was below our aim of 65%. However, the literature recognizes that among healthcare trainees, survey responses are more difficult to attain as postgraduate training level increases and when respondents do not work in the same institutions (while all trainees were part of the RCPI training program, they worked in different locations).^21,22^ Similarly, the study is limited by the smaller number of attending pediatricians that partook, and we could not estimate a response rate for this group. However, the fact that data were unavailable to calculate this also suggests a lack of attention to the issue. A strength is the fact that we were able to compare the opinion of both trainees and established pediatricians. It also could have been useful to compare the opinions of attendings working in larger units, but this was beyond the scope of the study. When considering recruitment barriers, the opinions of attending pediatricians were included, although we acknowledge this may introduce a survivorship bias.

## Conclusion

Many community units were established in the 1990s and consequently the retirements of many well-established community pediatricians are impending. We suggest several key points to improve retention and recruitment for these units. These include promoting skills necessary for generalist practice, improving environmental supports for pediatricians in community units, and providing supports for research and quality improvement. Unless action is taken, the existing staffing issues in these units are likely to worsen, potentially creating adverse health outcomes for children living in these underserved areas and increased demand for tertiary services.

## Supplemental Material

sj-docx-1-mde-10.1177_23821205241285599 - Supplemental material for Exploring the Factors That Impact Recruitment and Retention of Pediatricians in Irish Community Hospitals Through the Attitudes of Trainees and Physicians-in-Practice Supplemental material, sj-docx-1-mde-10.1177_23821205241285599 for Exploring the Factors That Impact Recruitment and Retention of Pediatricians in Irish Community Hospitals Through the Attitudes of Trainees and Physicians-in-Practice by Lydia Healy, Michael J O'Grady, Nigel Fancourt and Briseida Mema in Journal of Medical Education and Curricular Development

sj-docx-2-mde-10.1177_23821205241285599 - Supplemental material for Exploring the Factors That Impact Recruitment and Retention of Pediatricians in Irish Community Hospitals Through the Attitudes of Trainees and Physicians-in-Practice Supplemental material, sj-docx-2-mde-10.1177_23821205241285599 for Exploring the Factors That Impact Recruitment and Retention of Pediatricians in Irish Community Hospitals Through the Attitudes of Trainees and Physicians-in-Practice by Lydia Healy, Michael J O'Grady, Nigel Fancourt and Briseida Mema in Journal of Medical Education and Curricular Development

sj-docx-3-mde-10.1177_23821205241285599 - Supplemental material for Exploring the Factors That Impact Recruitment and Retention of Pediatricians in Irish Community Hospitals Through the Attitudes of Trainees and Physicians-in-Practice Supplemental material, sj-docx-3-mde-10.1177_23821205241285599 for Exploring the Factors That Impact Recruitment and Retention of Pediatricians in Irish Community Hospitals Through the Attitudes of Trainees and Physicians-in-Practice by Lydia Healy, Michael J O'Grady, Nigel Fancourt and Briseida Mema in Journal of Medical Education and Curricular Development

sj-docx-4-mde-10.1177_23821205241285599 - Supplemental material for Exploring the Factors That Impact Recruitment and Retention of Pediatricians in Irish Community Hospitals Through the Attitudes of Trainees and Physicians-in-Practice Supplemental material, sj-docx-4-mde-10.1177_23821205241285599 for Exploring the Factors That Impact Recruitment and Retention of Pediatricians in Irish Community Hospitals Through the Attitudes of Trainees and Physicians-in-Practice by Lydia Healy, Michael J O'Grady, Nigel Fancourt and Briseida Mema in Journal of Medical Education and Curricular Development
