# Supplementary material for: Exploring the Factors That Impact Recruitment and Retention of Pediatricians in Irish Community Hospitals Through the Attitudes of Trainees and Physicians-in-Practice
Source: J Med Educ Curric Dev. 2024 Oct 8;11:23821205241285599. doi: 10.1177/23821205241285599 (PMC11468435; doi:10.1177/23821205241285599)
Supplement: sj-docx-3-mde-10.1177_23821205241285599 - Supplemental material for Exploring the Factors That Impact Recruitment and Retention of Pediatricians in Irish Community Hospitals Through the Attitudes of Trainees and Physicians-in-Practice [file sj-docx-3-mde-10.1177_23821205241285599.docx]

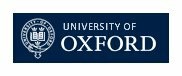


Survey about RCPI Trainees' Career Attitudes

# Page 1: Introduction

## Preparing Paediatricians for a career in Local Hospitals in Ireland CUREC Approval Reference: R85405/RE001

### General Information

We appreciate your interest in participating in this questionnaire. You have been invited to participate as you are a Higher Specialist Trainee in Paediatrics. Please read through this information before agreeing to participate (if you wish to).

The aim of this study is to investigate if existing Paediatric training pathways in Ireland could be altered to encourage more trainees to target a career as a general Paediatrician in a local unit. Local units as defined by the HSE Model of Care for Paediatrics include Letterkenny University Hospital, Sligo University Hospital, Mayo University Hospital, Portiuncula Hospital, Ballinasloe, Cavan Monaghan Hospital, Our Lady of Lourdes Hospital, Drogheda, Regional Hospital Mullingar, Midland Regional Hospital Portlaoise, St Luke's General Hospital Kilkenny, Wexford General Hospital, University Hospital Waterford, South Tipperary General Hospital and University Hospital Kerry. While the Mercy University Hospital is classed as a local unit, we will not consider it one for this survey as we are focusing on hospitals outside of major cities.

You may ask any questions before deciding to take part by contacting the researcher (details below). The Principal Researcher is Dr Lydia Healy who is attached to the Department of Education at the University of Oxford, where the overall supervisor is Nigel Fancourt, Associate Professor of Education and Values. Prof Michael O'Grady from RCPI is also providing supervision.

This survey will take approximately five minutes to complete. Questions relate to your career plans and general experience of training

### Do I have to take part?

No. Please note that participation is voluntary. If you do decide to take part, you may withdraw at any point for any reason before submitting your answers by closing the browser. However, once you submit the survey in full, as responses are anonymous, your data cannot be removed.

### Who will have access to my data and how will my data be used?

The University of Oxford and RCPI are the data controllers with respect to your Questionnaire about RCPI Trainees' Career Plans data and, as such, will determine how your data is used in the study. The University will process your personal data for the purpose of the research outlined above. Research is a task that we perform in the public interest. Further information about your rights with respect to your personal data is available from https://compliance.admin.ox.ac.uk/individual-rights.

The responses you provide will be stored in a password-protected electronic file on University of Oxford secure servers and may be used to inform Paediatric training design in the RCPI, as part of Lydia Healy's masters' thesis and they may be included in a journal publication. All data collected is anonymous and while we collect data such as your age range and gender (we are interested in these data points to ascertain any barriers faced due to same), we will not collect any data that could directly identify you.

### Who has reviewed this study?

This project has been reviewed by, and received ethics clearance through, a subcommittee of the University of Oxford Central University Research Ethics Committee and the Research Ethics Committee of the RCPI (references numbers R85405/RE001

and RCPI RESCAF 190).

### Who do I contact if I have a concern, or I wish to complain?

If you have a concern about any aspect of this study, please speak to Dr Lydia Healy or their supervisor Prof Nigel Fancourt (details below), and we will do our best to answer your query. We will acknowledge your concern within 10 working days and give you an indication of how it will be dealt with. If you remain unhappy or wish to make a formal complaint, please contact the Chair of the Medical Sciences Interdivisional Research Ethics Committee at the University of Oxford (details below) who will seek to resolve the matter as soon as possible.

## Thank you in advance

Lydia Healy, email: [lydia.healy@st-hildas.ox.ac.uk](mailto:lydia.healy@st-hildas.ox.ac.uk) Nigel Fancourt; [nigel.fancourt@education.ox.ac.uk](mailto:nigel.fancourt@education.ox.ac.uk)

Medical Sciences Interdivisional Research Ethics Committee; Email: ethics@medsci.ox.ac.uk; Address: Research Services, University of Oxford, Boundary Brook House, Churchill Drive, Headington, Oxford OX3 7GB


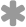
I confirm that I am over 18 years of age, I have read the information above and agree to participate with the understanding that the data I submit will be processed accordingly. *Required*


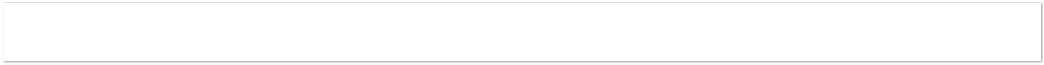

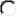


I agree

# Page 2: Demographics


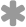
Please indicate your age *Required*


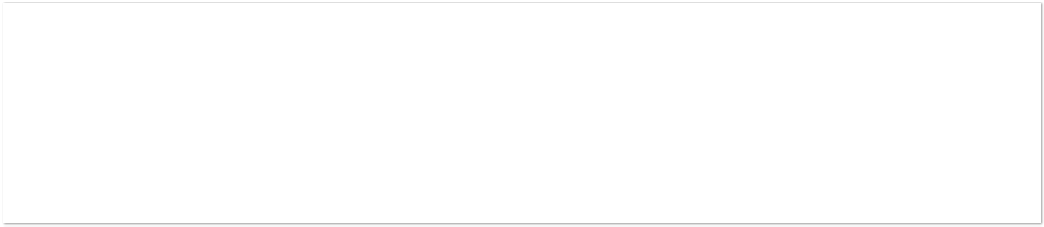

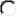

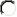

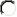

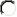

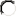

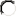


20-29

30-39

40-49

50-59

60-67

68 and older


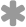
Please indicate your gender *Required*


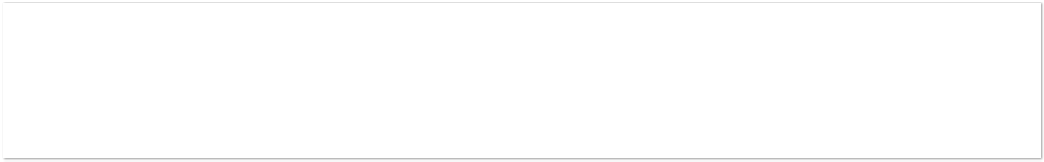

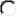

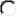

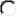

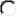


Male Female

Non-binary or other Prefer not to say

If you selected non-binary or other, and want to state your gender, please do:


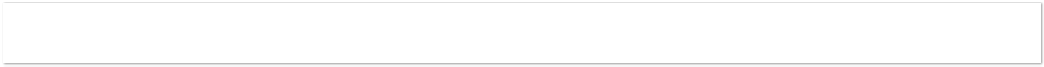


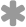
Please indicate your year of Higher Specialist Training. (If you are currently 'out of program' or on leave please indicate the most recent year you completed) *Required*


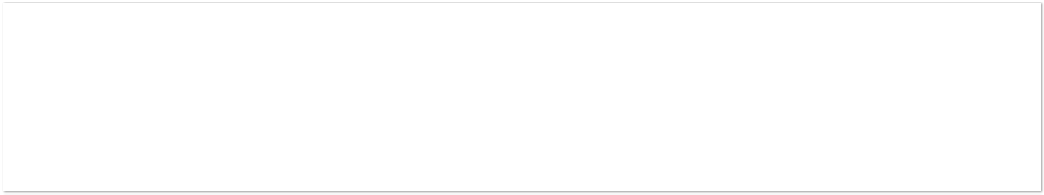

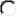

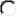

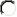

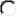

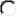


Year One Year Two Year Three Year Four Year Five

# Page 3: Career Plan

What is your current career aim after completing training in Paediatrics? *(General Paediatrician implies working on the General ‘on-call rota’ within any given hospital. Subspecialist Paediatrician implies working only on a subspecialist on-call rota e.g.*


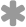
*Paediatric Gastroenterologist doing only gastroenterology on-call) Required*


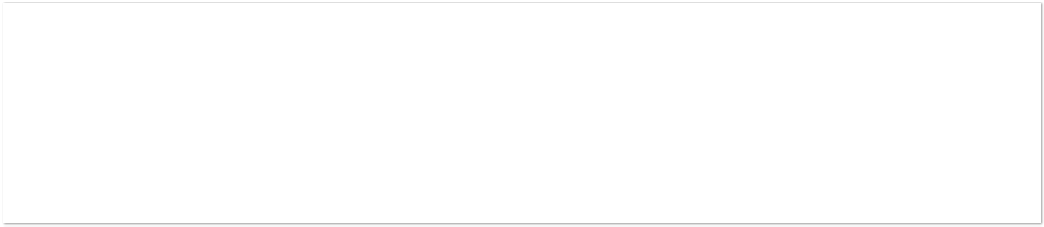

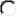

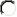

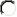

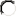

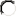

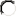


General Paediatrician

General Paediatrician with a Special Interest Subspecialist Paediatrician (including neonatology) I don't know yet

I don't plan to work in Paediatrics long-term Other

If you selected Other, please specify:


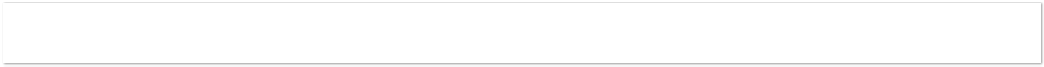


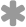
If you selected general paediatrician with a special interest for question 5, please indicate your preferred special interest

*Required*


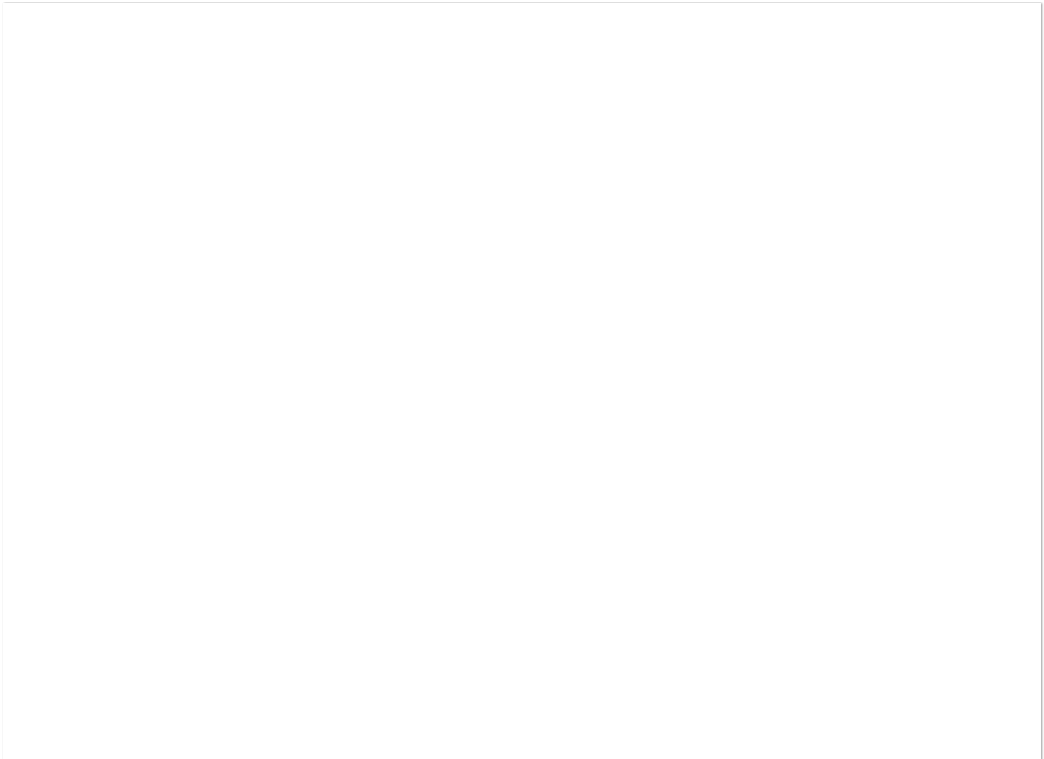

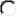

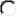

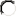

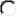

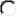

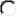

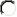

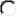

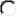

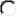

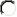

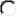

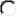

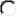

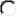

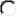

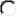

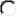

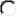

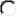

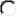

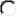

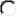


not applicable Respiratory Medicine Neurology Cardiology Gastroenterology Neurodisability

Child Protection Nephrology Endocrinology Metabolic Medicine Neonatology Rheumatology Allergy

Infectious Diseases Immunology Adolescent Medicine Mental Health Community Paediatrics Emergency Medicine Intensive Care Haematology Palliative Care

Other


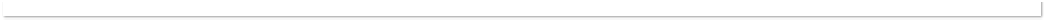


If you selected Other, please specify:


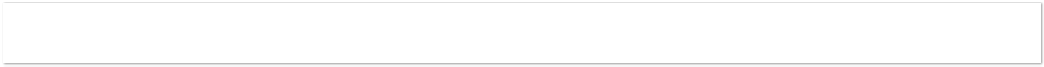


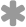
If you selected Subspecialist Paediatrician for question 5, please indicate your preferred specialism *Required*


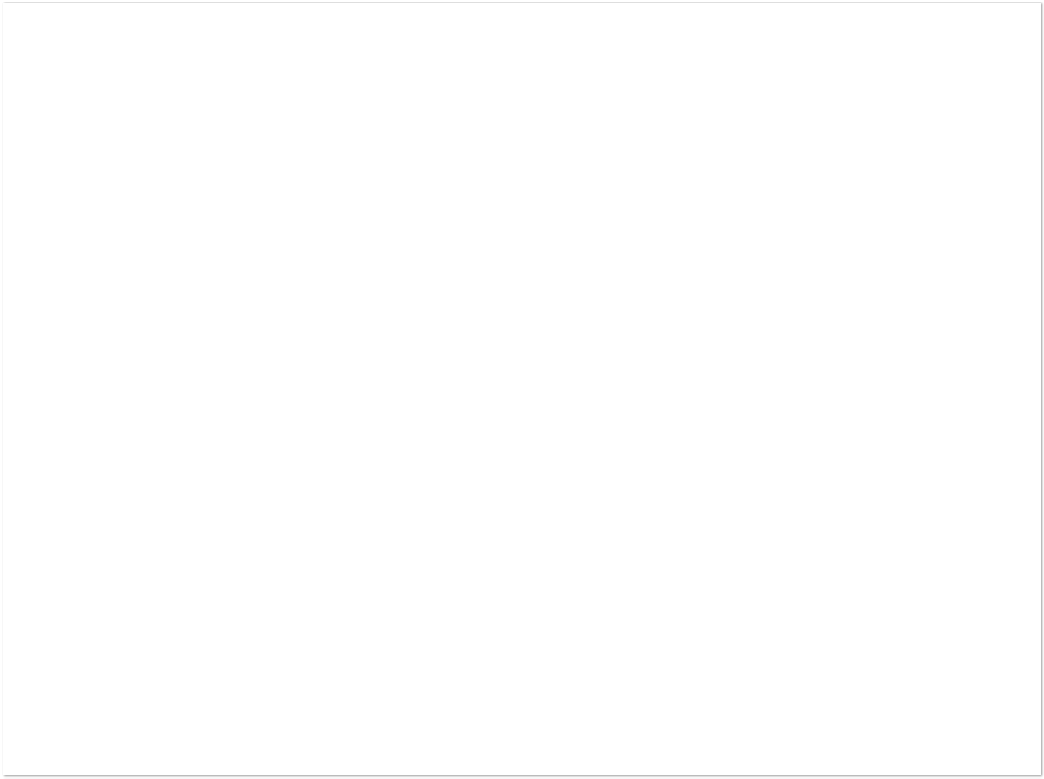

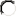

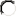

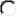

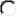

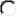

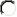

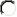

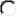

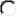

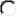

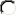

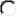

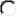

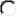

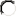

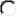

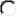

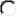

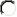

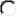

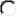

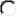

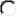


not applicable Respiratory Medicine Neurology Cardiology Gastroenterology Neurodisability

Child Protection Nephrology Endocrinology Metabolic Medicine Neonatology Rheumatology Allergy

Infectious Diseases Immunology Adolescent Medicine Mental Health Community Paediatrics Emergency Medicine Intensive Care Haematology Palliative Care

Other

If you selected Other, please specify:


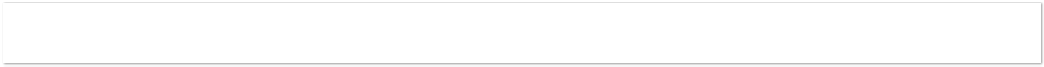


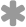
When did you decide on your current career plan? *Required*


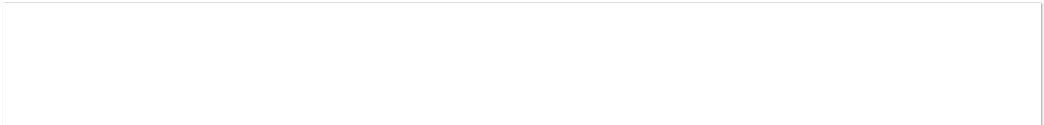

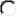

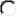

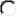


Before applying to HST After applying to HST

I don't have a career plan at the moment


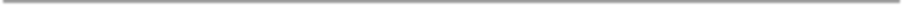


# Page 4: Location of Work


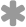
Where do you plan to work as a Paediatrician in the long-term? *Required*


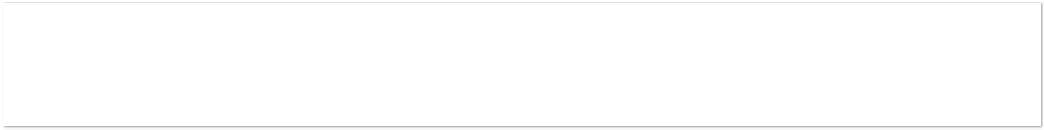

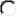

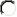

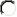


Republic of Ireland Northern Ireland

Outside of the island of Ireland

If you plan to work in Ireland, where do you hope to work? Please rank in order of preference. (CHI includes Crumlin, Temple Street, Blanchardstown, Tallaght and new St James’ campuses. Regional and Local Units are as defined in the Model of Care for Paediatrics and the Geographical options are listed below)

Please don't select more than 1 answer(s) per row. Please select at least 4 answer(s).

Please don't select more than 1 answer(s) in any single column.

|  | 1 | 2 | 3 | 4 |
| --- | --- | --- | --- | --- |
| Any CHI Hospital |  |  |  |  |
| Regional Paediatric Unit in a General Hospital in Cork, Limerick or Galway City |  |  |  |  |
| Local Paediatric Unit in a General Hospital in Letterkenny, Sligo, Mayo, Portiuncula, Cavan, Drogheda, Mullingar, Portlaoise, Kilkenny, Wexford, Waterford, Clonmel or Kerry |  |  |  |  |
| Maternity Hospital in any Irish location |  |  |  |  |

Regarding Paediatric Hospitals in Ireland, please indicate which hospitals you would be happy to work in as a consultant


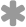
*please note, data relating to individual hospitals will not be published or shared outside of the RCPI

|  | *Required* | | |
| --- | --- | --- | --- |
|  | Yes | No | Undecided |
| Children's Health Ireland Hospitals (any) |  |  |  |
| Maternity Hospitals (any) |  |  |  |
| Galway University Hospital |  |  |  |
| University Hospital Limerick |  |  |  |
| Cork University Hospital |  |  |  |
| Mercy University Hospital, Cork |  |  |  |
| Letterkenny University Hospital |  |  |  |
| Sligo University Hospital |  |  |  |
| Mayo University Hospital, |  |  |  |
| Portiuncula Hospital, Ballinasloe |  |  |  |
| Cavan Monaghan Hospital |  |  |  |
| Our Lady of Lourdes Hospital, Drogheda |  |  |  |

| Regional Hospital Mullingar |  |  |  |
| --- | --- | --- | --- |
| Midland Regional Hospital Portlaoise |  |  |  |
| St Luke's General Hospital Kilkenny |  |  |  |
| Wexford General Hospital |  |  |  |
| University Hospital Waterford |  |  |  |
| South Tipperary General Hospital |  |  |  |
| University Hospital Kerry |  |  |  |

# Page 5: Working in a Local Paediatric Unit

Even if it is not your first choice, would you be open to working long-term in a Local Paediatric Unit? (i.e. Letterkenny University Hospital; Sligo University Hospital; Mayo University Hospital; Portiuncula Hospital, Ballinasloe; Cavan Monaghan

Hospital; Our Lady of Lourdes Hospital, Drogheda, Regional Hospital Mullingar; Midland Regional Hospital Portlaoise; St Luke's General Hospital Kilkenny; Wexford General Hospital; University Hospital Waterford; South Tipperary General Hospital; University Hospital Kerry) *Required*

Yes No

What are the reasons you would be open to working in a Local Paediatric unit? Please rank the options starting with the most important reason as no 1. You can select as many options as you want. *Required*

Please don't select more than 1 answer(s) per row. Please select at least 1 answer(s).

Please don't select more than 1 answer(s) in any single column.

|  | 1 | 2 | 3 | 4 | 5 | 6 | 7 | 8 | 9 |
| --- | --- | --- | --- | --- | --- | --- | --- | --- | --- |
| I come from a more rural background |  |  |  |  |  |  |  |  |  |
| Previous positive training experience |  |  |  |  |  |  |  |  |  |
| I want to be near family/friends |  |  |  |  |  |  |  |  |  |
| I perceive there is a better work/life balance |  |  |  |  |  |  |  |  |  |
| I perceive there is a better cost of living |  |  |  |  |  |  |  |  |  |
| I like the variety of work |  |  |  |  |  |  |  |  |  |
| The unit/units have a good reputation |  |  |  |  |  |  |  |  |  |

| It would be easier to secure a post without having to do additional training overseas |  |  |  |  |  |  |  |  |  |
| --- | --- | --- | --- | --- | --- | --- | --- | --- | --- |
| Not applicable; I am completely opposed to this option |  |  |  |  |  |  |  |  |  |
| Other |  |  |  |  |  |  |  |  |  |

If other please specify

What would discourage you from working in a Local Paediatric Unit? Please rank reasons in order of importance where 1 is the most important. You can choose as many as you want. *Required*

Please don't select more than 1 answer(s) per row. Please select at least 1 answer(s).

Please don't select more than 1 answer(s) in any single column.

|  | 1 | 2 | 3 | 4 | 5 | 6 | 7 | 8 | 9 | 10 |
| --- | --- | --- | --- | --- | --- | --- | --- | --- | --- | --- |
| A previous negative training experience |  |  |  |  |  |  |  |  |  |  |
| It would be too far from friends/family |  |  |  |  |  |  |  |  |  |  |
| It would not suit a spouse’s/partner’s career |  |  |  |  |  |  |  |  |  |  |
| On-call is more frequent |  |  |  |  |  |  |  |  |  |  |
| The reputation of the unit/units is not good |  |  |  |  |  |  |  |  |  |  |
| Some of my colleagues won’t have completed an approved training scheme |  |  |  |  |  |  |  |  |  |  |

| Reduced access to specialist services |  |  |  |  |  |  |  |  |  |  |
| --- | --- | --- | --- | --- | --- | --- | --- | --- | --- | --- |
| There is no on- site Paediatric Intensive Care |  |  |  |  |  |  |  |  |  |  |
| I do not want to manage Neonatal Emergencies |  |  |  |  |  |  |  |  |  |  |
| Some sites are not approved for SpR training |  |  |  |  |  |  |  |  |  |  |
| There is less infrastructure for research and/or service improvement |  |  |  |  |  |  |  |  |  |  |
| I don’t feel my training to date has prepared me for that type of consultant post |  |  |  |  |  |  |  |  |  |  |
| Nothing would discourage me from this type of work |  |  |  |  |  |  |  |  |  |  |
| Other |  |  |  |  |  |  |  |  |  |  |

If other please specify

Regarding neonatal experience, currently would you feel prepared to lead neonatal resuscitation in a local Paediatric Unit?

*Required*

Yes No

How much neonatal experience do you currently have? *Required*

I have at least 6 months’ experience of tertiary neonatology at registrar level

I have less than 6 months’ experience of tertiary neonatology at registrar level

Would any of the following make you significantly more likely to pursue a career as a Paediatrician in a local unit? Please rank

anwers in order of importance and choose as many as you want. *Required*

Please don't select more than 1 answer(s) per row. Please select at least 1 answer(s).

Please don't select more than 1 answer(s) in any single column.

|  | 1 | 2 | 3 | 4 | 5 | 6 | 7 |
| --- | --- | --- | --- | --- | --- | --- | --- |
| Dedicated training pathways |  |  |  |  |  |  |  |
| Shorter overall training time |  |  |  |  |  |  |  |
| More special interest fellowships in Ireland aimed at this career pathway |  |  |  |  |  |  |  |
| More opportunities to ‘act-up’ as consultant in local units |  |  |  |  |  |  |  |
| Financial incentives at consultant level |  |  |  |  |  |  |  |
| Nothing would make me more likely to pursue this career path |  |  |  |  |  |  |  |
| Other |  |  |  |  |  |  |  |

If other please specify

# Page 6: Final page

Thank you for taking part in the survey. If you are interesting in taking part in an additional interview as part of this research, please contact Lydia Healy at

- [lydia.healy@st-hildas.ox.ac.uk](mailto:lydia.healy@st-hildas.ox.ac.uk) or 0838001353
