# Supplementary material for: Exploring the Factors That Impact Recruitment and Retention of Pediatricians in Irish Community Hospitals Through the Attitudes of Trainees and Physicians-in-Practice
Source: J Med Educ Curric Dev. 2024 Oct 8;11:23821205241285599. doi: 10.1177/23821205241285599 (PMC11468435; doi:10.1177/23821205241285599)
Supplement: sj-docx-4-mde-10.1177_23821205241285599 - Supplemental material for Exploring the Factors That Impact Recruitment and Retention of Pediatricians in Irish Community Hospitals Through the Attitudes of Trainees and Physicians-in-Practice [file sj-docx-4-mde-10.1177_23821205241285599.docx]

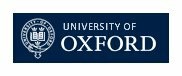


Survey for Paediatric Consultants working in Local Units

# Page 1: Introduction

## Preparing Paediatricians for a career in Local Hospitals in Ireland CUREC Approval Reference: R85405/RE001

### General Information

We appreciate your interest in participating in this questionnaire. You have been invited to participate as you are a **Paediatric consultant working in a Local Paediatric Unit in Ireland**. Please read through this information before agreeing to participate (if you wish to). The aim of this study is to investigate if existing Paediatric training pathways in Ireland could be altered to encourage more trainees to target a career as a General Paediatrician in a Local Unit.

Local units as defined by the HSE Model of Care for Paediatrics include Letterkenny University Hospital, Sligo University Hospital, Mayo University Hospital, Portiuncula University Hospital, Ballinasloe, Cavan Monaghan Hospital, Our Lady of Lourdes Hospital, Drogheda, Regional Hospital Mullingar, Midland Regional Hospital Portlaoise, St Luke's General Hospital Kilkenny, Wexford General Hospital, University Hospital Waterford, South Tipperary General Hospital and University Hospital Kerry. We have not included the Mercy Hospital in Cork as we want to focus on units outside of major cities. Please only respond to this survey if a **significant proportion of your practice** is in one of these units.

You may ask any questions before deciding to take part by contacting the researcher (details below). The Principal Researcher is Dr Lydia Healy who is attached to the Department of Education at the University of Oxford, where the overall supervisor is Nigel Fancourt, Associate Professor of Education and Values. Prof Michael O'Grady from RCPI is also providing supervision.

This survey will take approximately five minutes to complete. Questions relate to your career experience and opinions about current Paediatric training.

### Do I have to take part?

No. Please note that participation is voluntary. If you do decide to take part, you may withdraw at any point for any reason before submitting your answers by closing the browser. However, once you submit the survey in full, as responses are anonymous, your data cannot be removed.

### Who will have access to my data and how will my data be used?

The University of Oxford and RCPI are the data controllers with respect to your personal data and, as such, will determine how your personal data is used in the study. The University will process your personal data for the purpose of the research outlined above. Research is a task that we perform in the public interest. Further information about your rights with respect to your personal data is available from https://compliance.admin.ox.ac.uk/individual-rights.

The responses you provide will be stored in a password-protected electronic file on University of Oxford secure servers and may be used to inform Paediatric training design in the RCPI, as part of Lydia Healy's masters' thesis and they may be included in a journal publication. All data collected is anonymous and while we collect data such as your age range and gender (we are interested in these data points to ascertain any barriers faced due to same), we will not collect any data that could directly identify you.

### Who has reviewed this study?

This project has been reviewed by, and received ethics clearance through, a subcommittee of the University of Oxford Central University Research Ethics Committee and the Research Ethics Committee of the RCPI (references numbers R85405/RE001 and RCPI RESCAF 190).

### Who do I contact if I have a concern, or I wish to complain?

If you have a concern about any aspect of this study, please speak to Dr Lydia Healy or their supervisor Prof Nigel Fancourt (details below), and we will do our best to answer your query. We will acknowledge your concern within 10 working days and give you an indication of how it will be dealt with. If you remain unhappy or wish to make a formal complaint, please contact the Chair of the Medical Sciences Interdivisional Research Ethics Committee at the University of Oxford (details below) who will seek to resolve the matter as soon as possible.

## Thank you in advance

Lydia Healy, email: [lydia.healy@st-hildas.ox.ac.uk](mailto:lydia.healy@st-hildas.ox.ac.uk) Nigel Fancourt; [nigel.fancourt@education.ox.ac.uk](mailto:nigel.fancourt@education.ox.ac.uk)

Medical Sciences Interdivisional Research Ethics Committee; Email: ethics@medsci.ox.ac.uk; Address: Research Services, University of Oxford, Boundary Brook House, Churchill Drive, Headington, Oxford OX3 7GB


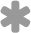
I confirm that I am over 18 years of age, have read the information above and agree to participate with the understanding that the data I submit will be processed accordingly. *Required*


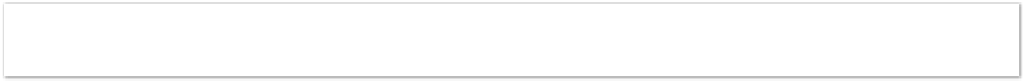

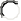


I agree

# Page 2: Demographics


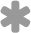
Please indicate your age-range *Required*


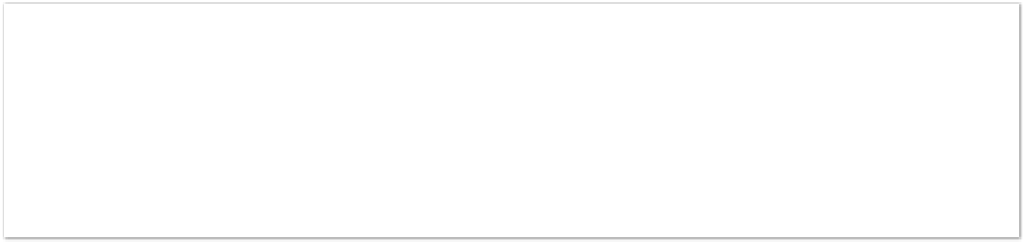

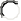

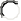

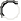

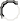

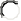


30-39

40-49

50-59

60-69

70 and older


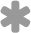
Please indicate your gender *Required*


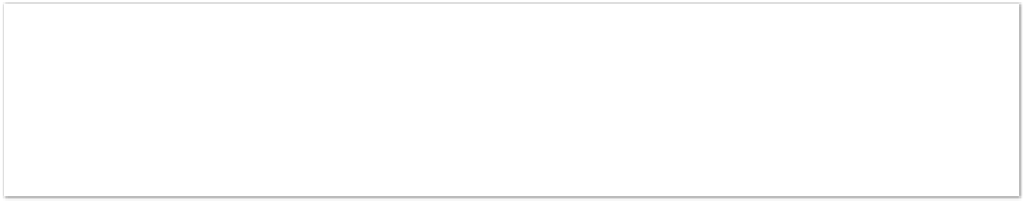

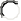

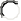

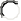

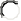


Male Female

Non-binary or other Prefer not to say

If you selected non-binary other, and want to state your gender please do:


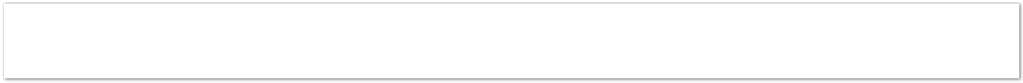


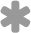
How long have you worked as a Paediatric consultant in Ireland? *Required*


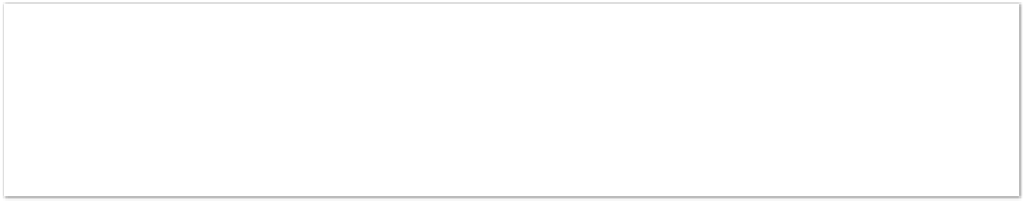

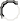

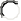

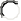

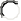


0-5 years

5-10 years

10-15 years

More than 15 years


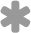
Please specify your medical council registration status *Required*


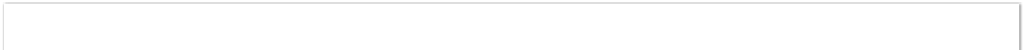

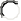


Specialist Division (Paediatrics)


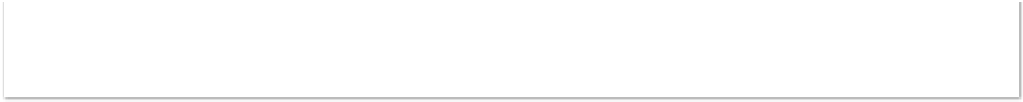

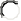

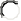


General Division Other

If you selected Other, please specify:


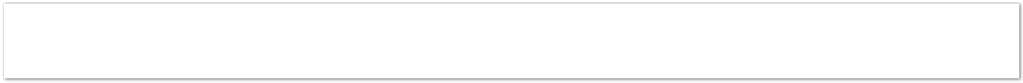


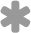
Where did you complete the majority of your postgraduate training in Paediatrics? *Required*


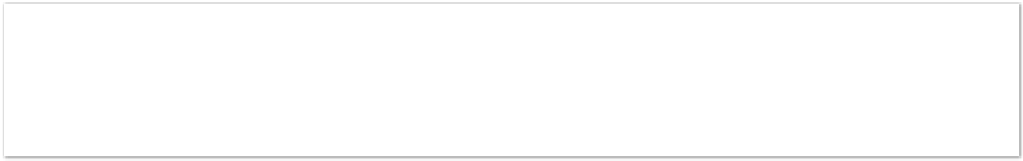

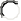

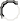

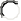


Exclusively in Ireland Mainly in Ireland Mainly Overseas


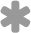
What is your current role *Required*


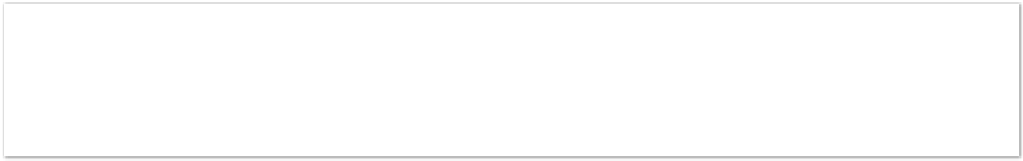

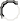

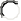

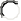


General Paediatrician

General Paediatrician with a special interest Other

If you selected Other, please specify:


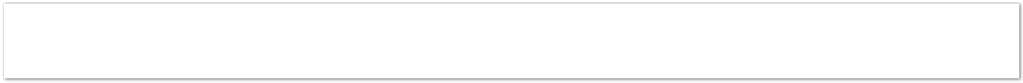


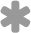
How are you currently employed *Required*


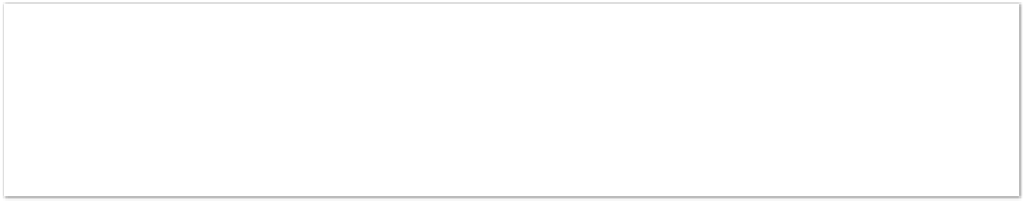

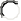

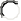

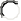

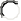


Permanent consultant contract Temporary/fixed-term consultant contract Locum or agency work

Other

If you selected Other, please specify:


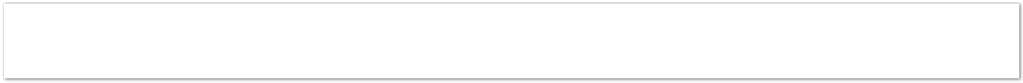


# Page 3: Working in a Local Paediatric Unit


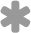
Why did you choose to work in your current hospital/unit? Please rank as many reasons as possible in order of importance (with 1 bring the most important) *Required*

Please don't select more than 1 answer(s) per row. Please select at least 2 answer(s).

Please don't select more than 1 answer(s) in any single column.

|  | 1 | 2 | 3 | 4 | 5 | 6 | 7 | 8 |
| --- | --- | --- | --- | --- | --- | --- | --- | --- |
| I had previous positive training experience in this/similar unit |  |  |  |  |  |  |  |  |
| To be near family/friends |  |  |  |  |  |  |  |  |
| Work/life balance |  |  |  |  |  |  |  |  |
| Cost of living |  |  |  |  |  |  |  |  |
| Job description suited my training |  |  |  |  |  |  |  |  |
| Good reputation of a unit |  |  |  |  |  |  |  |  |
| It was the job that was available |  |  |  |  |  |  |  |  |
| Other |  |  |  |  |  |  |  |  |

If other please specify


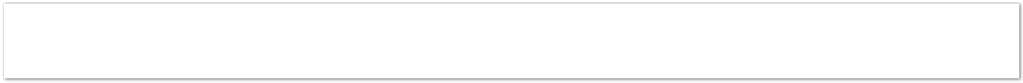


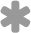
Do you feel current Paediatric training prepares trainees to work in a unit such as yours?

*Required*


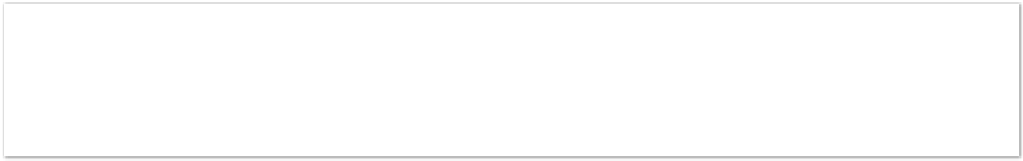

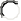

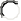

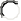


Yes No

Not sure

Please add any comments you may have


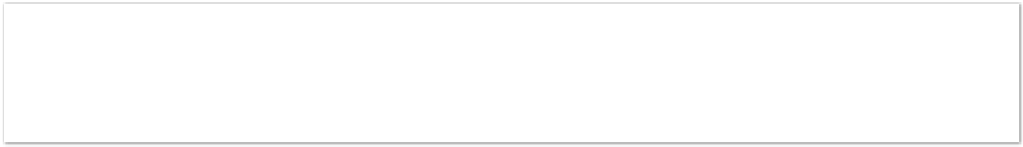


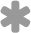
How do you think working in a Local Paediatric unit differs from working in a Regional or Tertiary unit? Please tick all that apply *Required*


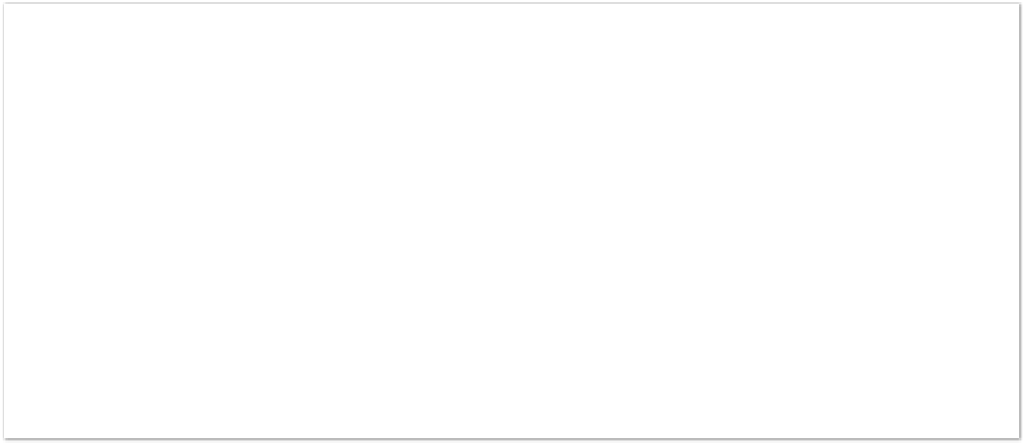


More autonomy More responsibility

More involvement in community Less variety in work

More variety in work

More exposure to resuscitation/emergencies Less support from other specialities

More opportunity to use specialist knowledge Less opportunity to use specialist knowledge Other

If you selected Other, please specify:


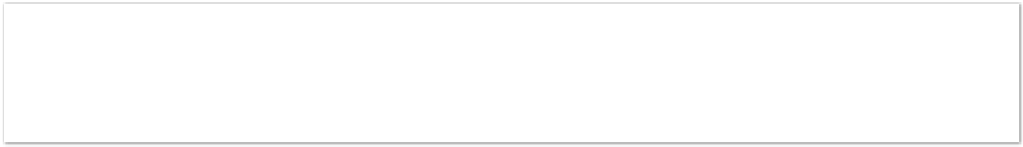


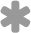
What do you think would be the best way to encourage more trainees to pursue a career as a paediatrician in a local unit? Please rank as many options as possible in order of importance *Required*

Please don't select more than 1 answer(s) per row. Please select at least 1 answer(s).

Please don't select more than 1 answer(s) in any single column.

|  | 1 | 2 | 3 | 4 | 5 |
| --- | --- | --- | --- | --- | --- |
| Dedicated training pathways |  |  |  |  |  |
| Shorter training time |  |  |  |  |  |
| More special interest fellowships in Ireland |  |  |  |  |  |
| Opportunities to ‘act- up’ as consultant |  |  |  |  |  |
| Financial incentives |  |  |  |  |  |
| Other |  |  |  |  |  |

If other please specify


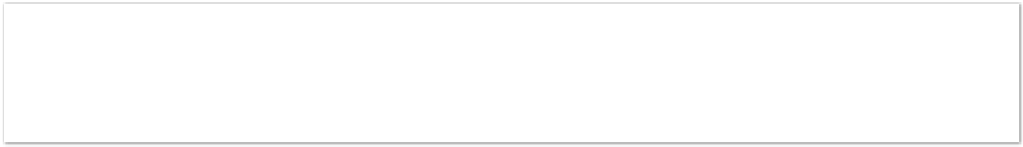


Please add any additional comments on this topic you may have


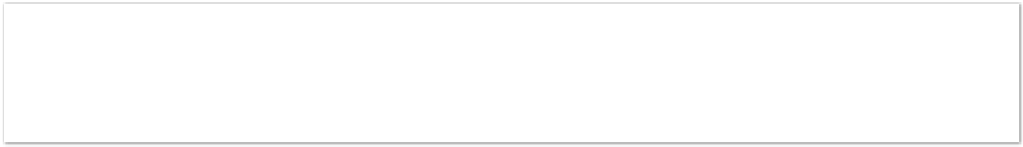


# Page 4: Final page

Thank you for taking part in the survey. If you are interesting in taking part in an additional interview as part of this research, please contact Lydia Healy at

- [lydia.healy@st-hildas.ox.ac.uk](mailto:lydia.healy@st-hildas.ox.ac.uk) or 0838001353
